# Dietary SCFAs, IL-22, and GFAP: The Three Musketeers in the Gut–Neuro–Immune Network in Type 1 Diabetes

**DOI:** 10.3389/fimmu.2019.02429

**Published:** 2019-10-29

**Authors:** Abhirup Jayasimhan, Eliana Mariño

**Affiliations:** ^1^Department of Immunology and Pathology, Monash University, Melbourne, VIC, Australia; ^2^Infection and Immunity Program, Department of Biochemistry, Biomedicine Discovery Institute, Monash University, Melbourne, VIC, Australia

**Keywords:** SCFA (short chain fatty acids), GFAP—glial fibrillary acidic protein, gut microbiota, glial cell, interleukin 22 (IL-22), ILC3s, beta cells, diabetes

## Abstract

Microbial metabolites have a profound effect on the development of type 1 diabetes (T1D). The cross-talk between the gut microbiota, the nervous system, and immune system is necessary to establish and maintain immune and gut tolerance. As quoted by Hippocrates, “All disease begins in the gut.” Although this has been recognized for 2,000 years, the connection between the gut and autoimmune T1D is not yet well-understood. Here, we outline new advances supported by our research and others that have contributed to elucidate the impact of microbial metabolites on the physiology of the pancreas and the gut through their remarkable effect on the immune and nervous system. Among many of the mechanisms involved in the gut–beta-cell–immune cross-talk, glial fibrillary acidic protein (GFAP)-expressing cells are critical players in the development of invasive insulitis. Besides, this review reveals a novel mechanism for microbial metabolites by stimulating IL-22, an essential cytokine for gut homeostasis and beta-cell survival. The close connections between the gut and the pancreas are highlighted through our review as microbial metabolites recirculate through the whole body and intimately react with the nervous system, which controls essential disorders associated with diabetes. As such, we discuss the mechanisms of action of microbial metabolites or short-chain fatty acids (SCFAs), IL-22, and GFAP on beta-cells, gut epithelial cells, neurons, and glial cells via metabolite sensing receptors or through epigenetic effects. The fine-tuned gut–neuro–immune network may be profoundly affected by SCFA deficiency related to dysbiosis and diet alterations at very early stages of the initiation of the disease. Thus, dampening the initial immune response or preventing the perpetuation of the immune response by maintaining the integrity of the gut is among the alternative approaches to prevent T1D.

## Introduction

Type 1 diabetes (T1D) is a chronic autoimmune disease in which T cells destroy the insulin-producing beta-cells of the pancreas (1–3). The beta-cell's attack happens when T cells recognize autoantigens such as glutamic acid decarboxylase (GAD), islet cell autoantigen 69 (ICA69), insulinoma-associated antigen 2 (IA2), islet-specific glucose-6-phosphatase catalytic subunit–related protein (IGRP), and proinsulin, which are widely accepted as the initiating autoantigens in T1D (4, 5). Antigenic targets for T cell priming are not solely expressed in beta-cells, but also in multiple tissues distal to islets, and they can be found in food like insulin or produced by bacteria like GAD (6–8). This all leads to many questions. How antigen expression in other distal tissues to beta-cells control the invasive infiltration of immune cells into the pancreas? Is the gut an important compartment as a source of antigens that trigger T1D? Is the gut microbiota influencing T cell priming against beta-cells? The microenvironment regulates beta-cell function and maturity, in particular close interaction with endocrine cells, neuronal, immune, and vascular cells (9, 10). Pancreatic ducts are physiologically neighboring to the beta-cells, and their primary function is to deliver enzymes or pancreatic juices provided from the exocrine pancreas into the duodenum to help digestion. As such, the pancreatic beta-cells can be influenced by the gut, which is intimately connected not only through the pancreatic ducts but also by lymph ducts (11). Beyond the pancreas, there is hardly any tissue in the body that has not been somehow in contact with gut microbial SCFAs. From food fermentation, bacteria in the large bowel produce many metabolites that are used by the epithelial cells in the gut. The remaining amount is transported to the liver where they are metabolized and then released to systemic circulation. As such, SCFAs have a broad spectrum of remarkable beneficial properties that affect many systems, in particular under inflammatory conditions, regulating metabolic, and immune responses (12–14).

One example is the nervous system, which is also critical for the pancreas to function (15). Both the endocrine and the exocrine part of the pancreas are innervated by the sympathetic and the parasympathetic nervous system, as such pancreatic sensory neurons have been shown to play a critical role in controlling islet inflammation (16). Similarly, the enteric nervous system (ENS) via the enteric glial cells (EGCs) is vital to maintain gut and immune homeostasis (17, 18), given that diabetic animals and patients presented gastrointestinal motility disorders (19). In this review, we will discuss the gut–neuro–immune axis in T1D and its effect on beta-cell priming. In particular, we will focus on the role of GFAP-positive cell types as critical players in T1D and on the impact of the gut microbiota, SCFAs, and their mechanisms of action through interleukin 22 (IL-22).

## GFAP—not the Usual Suspect!

Beta-cells are involved in late T cell priming, suggesting that they are not required during the induction of T1D (20, 21). So, a critical consideration is—what might be driving the initiation of T1D? It has been shown in the pancreas that GFAP-expressing peri-islet Schwann cells (pSC or glial cells) can attract and recruit autoreactive cells, which precedes the attack on beta-cells. Two studies support the finding that immune responses to autoantigens expressed in pSCs precede the immune response to beta-cells (6, 7). In particular, they showed that early T cell attack on GFAP-expressing pSCs progressively results in the release of glial cell antigens, GFAP, and insulin (6, 7). GFAP epitopes for autoreactive T and B cells have now been identified in non-obese diabetic (NOD) mice and humans with T1D. Serum GFAP antibodies are now used as a predictive marker for the development of T1D, and it has been shown that administration of GFAP as a vaccine delayed the progression of T1D by regulating T cell differentiation (22, 23). GFAP-expressing glial cells of the peripheral nervous system require TRPV1 expression for their proper maturation, and studies have shown that depleting TRPV1-expressing cells reduced the development of insulitis in NOD mice (16, 24). It is fascinating that a cytoskeletal protein widely expressed in pancreatic ductal cells and also in pancreatic glial cells of the central and peripheral nervous system may work as an early autoantigen in T1D.

Exploring further this idea, Slattery's group has recently shown that ablation of autoantigen presentation in GFAP-expressing cell types reduced the development of invasive insulitis in NOD mice (25). We can speculate that reduction, but not total elimination of invasive infiltration, may be due to the absence of presentation of autoantigens other than insulin by GFAP-expressing cells, suggesting that Ag-derived ductal cell is one of the critical requirements in orchestrating the initiation of autoimmune responses to beta-cell antigens.

## The SCFAs: Modulator of Gut Inflammation and Autoreactivity

After many years of efforts and studies focusing on the destruction of the beta-cells in the pancreas, there is still no cure or method of prevention for T1D. So, it makes us wonder whether we have been losing the battle only because we are not looking beyond the walls of the pancreas. T1D can be viewed as an orchestrated autoimmune response originated in the gut. This is evident from the observation that in many autoimmune diseases including T1D, the integrity of the epithelial barrier is compromised, leading to a phenomenon termed as “leaky gut” (26, 27). Pathogens, microbial products, and food-derived antigens find the leaky gut as a route to encounter the resident immune cells. For example, Gram-negative bacteria produce lipopolysaccharides (LPS), an identified endotoxin that can induce immune responses via the toll-like receptor 4 (TLR-4) expressed on monocytes (28). Given the gut connects to the pancreas through pancreatic lymph nodes (PLNs) and mesenteric lymph nodes (MLNs), bacterial and food products can hyperactivate resident T and B autoreactive cells in the gut or the gut-associated MLNs (29). Alternatively, it has been shown that gut microbial products can reach PLNs and locally modify the presentation of pancreatic self-antigens (30). Therefore, excess of food, chemicals, and microbial antigens can skew the intestinal immune system toward a perpetually pro-inflammatory state that may trigger T1D. Newly-diagnosed children with T1D present autoantibodies to GAD, a pancreatic beta-cell autoantigen that is also produced by many bacterial species (31). For instance, T1D patients present antibodies against a heat shock protein from the *Mycobacterium avium* subspecies *paratuberculosis*, MAP Hsp65, which has a high degree of homology with human GAD65, suggesting that cross-reactivity between MAP Hsp65 and GAD65 potentially could be a mechanism of triggering TID (32). Strong homology has been found between the islet-antigen IGRP and several gut- and oral-derived microbial peptides. These peptide sequences encode for magnesium transporter (Mgt), for hypothetical protein IEM_00289 and NAD synthetase, respectively, which activate NY8.3 CD8^+^ T cells with comparable potency to IGRP native peptide (33). Thus, molecular mimicry between excess of gut microbial antigens and islet cell autoantigens may be a mechanism by which gut dysbiosis leads to T1D development.

T1D is a multifactorial condition; diet and environment play an inevitable role in disease modulation (1, 13). Human and murine studies have demonstrated that defects in the induction of central and peripheral tolerance checkpoints (34) also correlate with an altered gut microbiota (35–39), which are notable contributors to T1D pathology. Building on previously extensive reviews on the gut microbiota topic, we have firmly discussed that an altered microbiota and SCFA deficiency are primary causal factors triggering T1D (12–14, 40). The gut microbiota through the production of dietary SCFAs plays a significant role in host defense by modulating the immune system and metabolism. Studies conducted by our group have shown that the combination of a diet rich in acetate and butyrate protected 90% of the NOD mice against T1D, yielding exceptionally high levels of the corresponding SCFAs to the feces (35). In this study, SCFA-induced T1D protection happened via changes in gut/immune regulation-expanding regulatory T (Treg) cells and reducing pathogenic B cells, CD4^+^, and CD8^+^ T cells. Diet rich in SCFA acetate and butyrate not only reduced the levels of serum LPS and pro-inflammatory interleukin 21 (IL-21) but also increased the level of serum IL-22, an important cytokine that maintains a healthy commensal microbiota, gut epithelial integrity, and mucosal immunity and ameliorates metabolic disease (41–44). Alternatively, SCFAs can also reduce islet-specific immune responses by increasing the production of antimicrobial peptides (AMPs) in the beta-cells (45). As it has been shown, C-type lectin regenerating islet-derived protein IIIγ (REGIIIγ) and defensins disrupt surface membranes of bacteria, thus enabling a broad regulation of commensal and pathogenic bacteria in the gut (46–48). Diana's group showed that insulin-secreting beta-cells produced the cathelicidin-related antimicrobial peptide (CRAMP), which was defective in NOD mice. Intraperitoneal administration of SCFA butyrate stimulates the production of CRAMP on pancreatic beta-cells via G protein-coupled receptors (GPCRs), which also correlated with the conversion of inflammatory immune cells to a regulatory phenotype (45). Likewise, another study has shown that microbial SCFAs contribute to the increasing concentrations of serum IL-22 (35) required for beta-cell regeneration by up-regulating the expression of Regenerating Reg1 and Reg2 genes in the islets (49).

There are pieces of evidence of compromised gut integrity, dysbiosis, and associated inflammation of the gastrointestinal tract (GI) in NOD mice and patients with T1D (50–55), similar to what has been shown in other inflammatory or autoimmune gut diseases (i.e., infection, celiac disease, IBD). The gut microbiota and the ENS play a critical role in diabetic gastrointestinal motility disorders, as individuals with diabetes suffer from symptoms such as nausea, heartburn, vomiting, diarrhea, abdominal pain, and constipation (56, 57). For example, it is known that slow motility of the GI leads to alterations of the gut microbiota that favors pathogenic bacterial overgrowth and subsequently diarrhea (58, 59). As such, the abundance and diversity of bacteria needed to maintain the integrity of the gut were significantly lower in children with T1D compared to healthy controls (60). On the other hand, animal studies have suggested that accelerated colonic transit time, relative to constipation, could be caused by autonomic neuropathy and diabetes-induced denervation of sympathetic nerve terminals (56, 61). Diet and/or deficiency of dietary SCFAs can also modulate the intestinal motility and survival of enteric neurons by miRNAs, which are involved in energy homeostasis, lipid metabolism, and proliferation and development of GI smooth muscles. miRNAs have been vastly studied in organ damage caused by diabetes, and one study has shown in mice that high-fat diets delay the GI transit, partly by inducing apoptosis in enteric neuronal cells, and this effect was shown to be mediated by Mir375 associated with reduced levels of Pdk (62). There is still too much to understand about the intrinsic mechanisms underlying the connection between the gut microbiota and the ENS and how this affects the course of T1D. Particularly high-fiber or specialized acylated starch diets that boost the microbial production of SCFAs are effective in the control of gut infections and diarrhea, as it has been shown to promote commensal acetate-producing bacteria (63).

## IL-22 and ENS Take Control of T1D

Activation of IL-22 through microbial SCFAs contribute to the maintenance of gut homeostasis by the close connection between the intestinal-resident innate lymphoid cell 3 (ILC3) and EGCs. IL-22 is expressed by ILC3, which lies close to EGCs (64), but its role in T1D is still elusive (14). ILCs sustain appropriate immune responses to commensals and pathogens at mucosal barriers by potentiating adaptive immunity and regulating tissue inflammation (65, 66). Likewise, EGCs have critical roles in maintaining gut homeostasis, as they can sense the pathogenic bacteria through toll-like receptors (TLRs). EGCs surround neurons and also connect with blood vessels and lymphatics (67), which allowed EGC-derived signaling molecules to modulate mucosal immunity. As such, EGCs sense environmental stimuli and extend their stellate projections into the ILC3 aggregates within the crypto-patches of the intestinal lamina propria and release neurotrophic factors that stimulate IL-22 secretion from ILC3s (68). The notion that gut microbiota affects the development and maturation of EGCs was shown in germ-free (GF) mice, which present a defective influx of EGCs into the intestinal mucosa (69). This occurs via expression of the neuroregulatory receptor (RET), as ablation of RET in ILC3 leads to reduced IL-22 production and compromised epithelial protection in colon inflammation mouse models (69).

Aligned with this idea, does the early autoreactivity to GFAP observed during insulitis originate in the gut? This is possible to the connections between the pancreas, the ENS, and the gut. The fine-tuned neuro–beta-cell cross-talk is more likely to be broken by the pathological changes occurring in a perturbed gut. Alterations of the gut microbiota, referred to as dysbiosis, decrease epithelial permeability, causing inflammation, and associated tissue damage that exposes numerous self-antigens harbored in the gut and associated enteric neuronal tissues. Gut microbial products can also sense enteric neurons and EGCs partly by pattern recognition receptors, such as TLRs. Indeed, pathogenic and commensal SCFA-producing bacteria up-regulate differentially toll-like receptor 2 (TLR2) expression on human EGCs (70). Expression of TLR2 on enteric neurons and EGCs controls nNOS^+^ neurons and acetylcholine-esterase-stained fibers in the myenteric ganglia. For example, *Escherichia coli* promoted expression of MHC II molecules on EGCs and significantly induced S100B protein overexpression and nitric oxide (NO) release from EGC, which was counteracted by pre-treatment with TLR and S100B inhibitors (70). As such, the myenteric plexus of TLR2Ko mice presented reduced expression of glial markers, GFAP, and S100B. Overexpression of GFAP has been observed to correlate with inflammatory responses in the gut (71). S100B is considered as a neurotrophin, due to its either tropic or toxic effects depending on the concentration in the extracellular milieu. Excess amount of S100B acts on RAGE (receptor for advanced glycation end-products), leading to the phosphorylation of mitogen-activated protein kinases (MAPK) and subsequent activation of the nuclear factor κB (NF-κB) and the associated release of NO. Excess NO causes damage to the tissue, resulting in inflammation and reduced integrity of the guts (72, 73). The protective role of EGCs in the maintenance of the gut epithelial integrity has been demonstrated in mice lacking GFAP-positive (+) glia that presented fatal hemorrhagic jejuno-ileitis (74).

During chronic tissue inflammation, significantly increased expression of GFAP on glial cells after stimulation with LPS and pro-inflammatory cytokines has been shown (75), similar to what has been seen in Crohn's disease (CD) and necrotizing enterocolitis (NEC). On the other hand, the presence of MHC class II expression on activated EGCs suggests that these cell types can present antigens (76, 77) derived from multiples sources, including microbes and host. EGCs, with the help of their stellate projections, sample microbial antigens crossing the epithelial barrier and activate diabetogenic T cells. This is given under dysbiosis, predominant in T1D and many autoimmune diseases, and the release of microbial antigens such as LPS may break the tolerance of EGCs leading to overexpression of glial cell markers GFAP and S100B. Thus, GFAP-expressing glial cells may have a protective role in maintaining the integrity of the gut, but under uncontrolled inflammatory conditions, it may lead to autoreactivity. As such, glial cell-derived protein GFAP is now an identified autoantigen in T1D and autoantibodies to GFAP has been detected in NOD mice and humans with T1D (23), thus showing the relevance of the microbiota–EGC pathways in T1D.

One study has shown that SCFA butyrate can induce increasing excitatory choline acetyltransferase (ChAT) neurons through the butyrate transporter monocarboxylate transporter (MCT), which is expressed by enteric neurons (78). However, it is still unknown what factors control neuronal MCT2 expression. Further studies will be necessary to determine how SCFAs regulate MCT2 expression and control the activity of intestinal neural circuits. SCFAs exert their function through two mechanisms, via metabolite sensing GPCRs or inhibition of histone deacetylase (HDAC) activity (13, 35, 79, 80). There are three receptors for SCFA acetate, butyrate, and propionate, namely GPR43 (FFA2), GPR41 (FFA3), and GPR109a. GPR43 is activated by SCFAs with varying potency—acetate > propionate > butyrate. GPR43 is expressed on gut epithelial cells and certain immune cells (81). GPR109a is expressed on a variety of immune cells, as well as adipocytes, hepatocytes, gut and retinal epithelium, vascular endothelium, and neuronal tissue (82). GPR109a is primarily activated by both niacin and butyrate ligands. While niacin levels are not high enough to activate the receptor under normal physiological conditions, levels of butyrate, obtained from the gut environment, and its oxidized form, β-hydroxybutyrate, are sufficient to stimulate a response (82). Similarly, GPR41 has been reported to be expressed on EGCs and enteric neurons (83, 84). GPR41 also binds the three major SCFAs, but with differing affinities (85).

Similar to the effects exerted through the GPCRs, SCFAs can influence the function and development of immune cells directly through epigenetic regulation of gene expression such as inhibition of HDACs (13, 86). HDACs allow the conversion of repressive chromatin structures, which takes place on lysine residues on N-terminal tails of histones 3 and 4, to increase gene transcription. HDACs are a group of 18 known enzymes that remove acetyl groups from the histones tails that bind DNA (87). Although little is known about the effects of SCFAs on EGCs through epigenetic modifications, it has been shown that butyrate treatment increases acetylation of the H3K9 in primary enteric neurons and the EGC *in vitro* (84).

**Figure 1:**
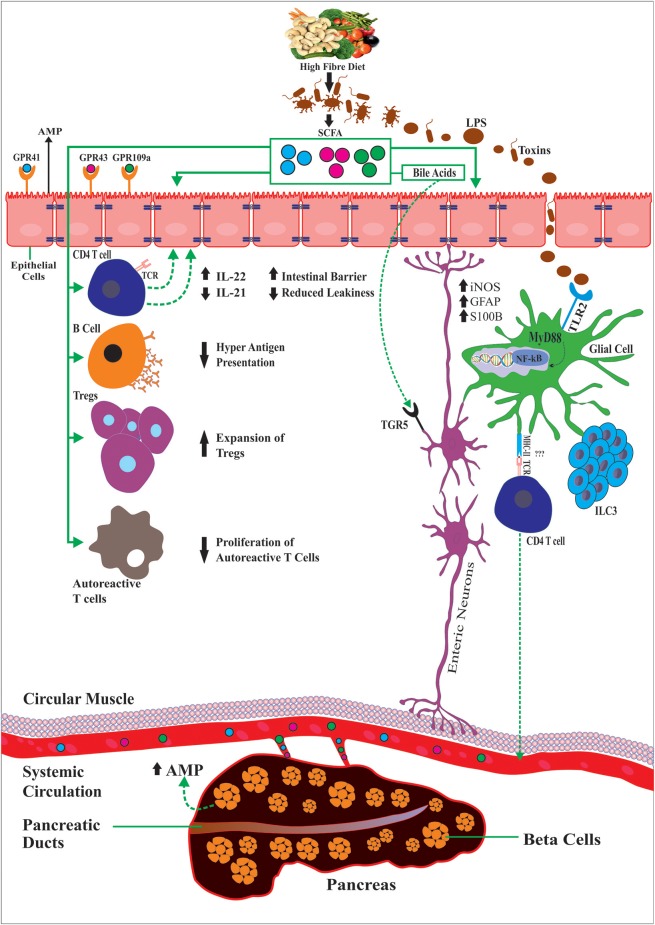
Diet and gut microbiota through the production of dietary SCFAs exert anti-inflammatory effects by controlling the activity of multiple immune cell types, outside or locally in the intestinal mucosa, the enteric glial cells and neurons but also glial cells in the pancreatic islets and the beta-cells. As such SCFAs promote IL-22 production CD4^+^ T cells or by supporting ILC3 cells, the major producers of IL-22. Also SCFAs can reduce production of pro-inflammatory cytokines IL-21, LPS, induce beta cell regeneration via AMPS, regulate GFAP in the gut and beta-cells, modulate the expansion of regulatory T and reduction of autoreactive CD8 T cells and reducing B cell hyperactive antigen presentation capacity. Activation of GPRCs (GPR41 and GPR43) on enteroendocrine cells of the intestinal epithelium and TLR signaling (e.g., TLR2 and TLR4) maintains subsets of enteric neurons resulting changes in gut motility, conversion of primary bile acids into secondary bile acids, which activate TGRS expressed by enteroendocrine cells and enteric neurons among many others.

SCFAs can also modulate gut motility by the production of serotonin by epithelial enterochromaffin cells (ECs) (88, 89). For instance, GF mice present gut dysmotility that was reversed by inoculation with SCFA-producing bacteria. Tested in human-derived EC cell lines, SCFAs increased serotonin (5-hydroxytryptamine [5-HT]) by up-regulating THER expression of tryptophan hydroxylase 1 (Tph1) (89) and by the serotonin-selective reuptake transporter (SERT), which is expressed by intestinal epithelial cells (90). Another critical role of SCFAs on the ENS is evidenced by the conversion of primary bile acids synthesized *de novo* into secondary bile acids in the liver (91). Aside from their role in dietary fat absorption, secondary bile acids can activate several GPCRs and nuclear hormone receptors, including the G-protein-coupled bile acid receptor 1 (TGR5) and farnesoid X receptor (FXR), highly expressed in enteric neurons and enteroendocrine L cells that improved intestinal inflammation and glucose tolerance in HFD-fed mice (92). TGR5 also affect peristalsis that is mediated partly by serotonin 5-HT (93), implicating its potential for the treatment of constipation and diarrhea. Altogether, this suggests the relevance of the gut–neuro–immune axis in T1D (Figure 1).

## Concluding Remarks

Among the described effects that SCFAs have on modulating the immune system, beta-cell biology, and gut homeostasis, we have uncovered a novel role for SCFAs by modulating the ENS in the gut, central for the control and prevention of T1D. Overall, an immune response to antigens presented not only by GFAP-expressing pSCs in the pancreas but also by GFAP-expressing EGCs in the gut is a novel finding involved in the initiation of the autoimmune process. Could it trigger antigen-experienced autoreactive cells to move up the gut and reach the ductal and beta-cells, and break the GFAP-expressing neuronal mantle of the islets? This is an unexplored field and requires further research. Given the close location and connection between the gut and the pancreas and their intrinsic dependence from the nervous system, this fine-tuned immuno–neuro-islet cross-talk may be profoundly affected by perturbed gut homeostasis at very early stages of the initiation of the T1D. Dampening the initial immune response or preventing the perpetuation of the islet-specific immune response by maintaining the integrity of the gut is among the possible therapeutic approaches to reprogram T1D (12, 14). Thus, any hope for a cure may lie in methods that can halt immune-mediated beta-cell damage by maintaining or improving gut–immune tolerance.

## Author Contributions

EM developed the conceptual idea, wrote and edited the manuscript. AJ wrote and edited the manuscript.

### Conflict of Interest

The authors declare that the research was conducted in the absence of any commercial or financial relationships that could be construed as a potential conflict of interest.
